# Extra-Intestinal Manifestations of Coeliac Disease in Children: Clinical Features and Mechanisms

**DOI:** 10.3389/fped.2019.00056

**Published:** 2019-03-05

**Authors:** Silvia Nardecchia, Renata Auricchio, Valentina Discepolo, Riccardo Troncone

**Affiliations:** ^1^Department of Medical Translational Sciences and European Laboratory for the Investigation of Food-Induced Diseases, University of Naples Federico II, Naples, Italy; ^2^Department of Medicine, University of Chicago, Chicago, IL, United States

**Keywords:** extraintestinal, celiac disease, children, gluten free diet, manifestation, clinical presentation, prognosis

## Abstract

Celiac disease (CD) is a systemic autoimmune disease due to a dysregulated mucosal immune response to gluten and related prolamines in genetically predisposed individuals. It is a common disorder affecting ~1% of the general population, its incidence is steadily increasing. Changes in the clinical presentation have become evident since the 80s with the recognition of extra-intestinal symptoms like short stature, iron deficiency anemia, altered bone metabolism, elevation of liver enzymes, neurological problems. Recent studies have shown that the overall prevalence of extra-intestinal manifestations is similar between pediatric and adult population; however, the prevalence of specific manifestations and rate of improvement differ in the two age groups. For instance, clinical response in children occurs much faster than in adults. Moreover, an early diagnosis is decisive for a better prognosis. The pathogenesis of extra-intestinal manifestations has not been fully elucidated yet. Two main mechanisms have been advanced: the first related to the malabsorption consequent to mucosal damage, the latter associated with a sustained autoimmune response. Importantly, since extra-intestinal manifestations dominate the clinical presentation of over half of patients, a careful case-finding strategy, together with a more liberal use of serological tools, is crucial to improve the detection rate of CD.

## Introduction

Celiac disease (CD) is a systemic autoimmune disease due to a dysregulated mucosal immune response to gluten and related prolamins, characterized by a remodeling of the small intestinal mucosa leading to villous atrophy (1), that recedes upon a gluten free diet (GFD). It occurs in genetically susceptible individuals carrying the HLA-DQ2 and/or -DQ8 and affects ~1% of the general population in Europe, North America, North Africa, India and Middle East (2). There is evidence that its incidence is steadily increasing (3), not only because of improved awareness and more extensive use of specific diagnostic tools. Genetic factors cannot explain such a rapid increment, hence it seems to be mainly attributable to environmental factors (4).

Concomitantly with the increase in CD incidence, changes in its clinical presentation have been described since the 80s. In contrast to the “typical” presentation of CD with gastrointestinal symptoms, a higher number of asymptomatic cases has been detected by targeted screening of at-risk groups (5), as well as an increase in the number of “atypical” presentations, including extra-intestinal symptoms such as iron deficiency anemia, altered bone metabolism, short stature, and elevation of liver enzymes. A recent American study showed that non-intestinal symptoms were the most commonly represented in 43% of pediatric CD patients (6). Importantly, the use of terms such as “typical” and “atypical” to describe the clinical presentation of CD, which reflects the historical background, is currently discouraged in favor of the use of “classical” when the gastrointestinal symptoms (such as weight loss, diarrhea, distended abdomen) are prominent and “not classical” indicating cases with a predominance of extra-intestinal symptoms (7).

The overall clinical picture of CD at diagnosis became less severe (8) and the average age at diagnosis increased from below 3 years to the scholar age (9). However, most of these changes seem to have recently reached a plateau, at least as reported in Finland (9).

Recent studies showed that the prevalence of extra-intestinal manifestations is similar between the pediatric and adult population: 60 and 62%, respectively (10). However, clinical manifestations and rate of improvement differ in the two age groups. Short stature was found to be the most common feature in children, while iron deficiency anemia dominates the clinical picture in adults, furthermore, clinical symptoms in children seem to recede much faster than in adults (10, 11).

Here we reviewed the current knowledge about the extra-intestinal manifestations of CD, operating a clear distinction between extra-intestinal symptoms and CD-associated conditions. Emphasis has been given to the mechanisms underlying these pleomorphic manifestations, even though in part still unclear. Finally, we analyzed the impact of the GFD on these extra-intestinal manifestations.

## Associated diseases vs. extra-intestinal manifestations

There is often some confusion between the extra-intestinal symptoms and diseases associated to CD. Indeed, some clinical presentations are sometimes described as CD-associated conditions and other times as extra-intestinal manifestations, one example being dermatitis herpetiformis. The main difference is that extra-intestinal symptoms improve on a GFD, particularly if the diet is started early (12). Contrariwise, CD-associated conditions are not correlated with gluten ingestion, despite being more frequent in the CD population. In line with what observed in many autoimmune disorders, there is an association of CD with other autoimmune conditions. The recent literature reports a risk of having another autoimmune disease, in the celiac population is from 3 to 10 times more frequent than in the general population (13, 14). The most frequent associated disease is type 1 diabetes (15), that shares with CD a combination of genetic factors and common pathogenetic mechanisms. The association with other autoimmune disorders is also attributed to shared genetic risk factors, particularly the HLA genes. In addition, a possible role of gluten in triggering autoimmunity has been suggested, based on its pro-inflammatory properties and the increased risk of developing other autoimmune disorders reported in relation to the duration of gluten containing diet (16). On the contrary, the pathogenic link of CD with chromosomal abnormalities such as Down's syndrome or Turner's disease is unclear and so is for other associated conditions such as IgA deficiency.

## Clinical manifestations

### Short Stature

After serological screening was introduced, in the form of anti-gliadin antibodies testing, the first extra-intestinal manifestation to be identified was short stature (17). Affecting ~10–40% of pediatric patients at the time of diagnosis (18), it still remains the most common extra-intestinal presentation of CD in children (19), sometimes being its only clinical sign (20). Up to 8% of the patients investigated for short stature will eventually receive a diagnosis of CD, that overall represents between 19 and 59% of all non-endocrinological causes of short stature (21–24). Poor growth has been more often described in children with a younger age at diagnosis and a more severe disease onset (25).

The pathogenic mechanisms underlying short stature in CD have not been fully clarified yet. Malnutrition due to malabsorption has traditionally been thought to play a major role, but more recently a multifactorial pathogenesis has been advanced. Particularly, dysfunction of the growth hormone (GH)- Insulin-like growth factor (IGF1) axis and in particular a role for ghrelin has been proposed (26). Reduced blood values of GH, IGF1, IGF-binding protein 1 and 3 (IGFBP 1, IGFBP3) and elevated levels of IGFBP2 have been reported (27). Interestingly, the exogenous administration of GH to untreated CD patients did not induce an increase in IGF-1 levels suggesting a dysfunction of growth axis associated to active CD (23). Dysregulation of the GH axis might be sustained by the elevation of pro-inflammatory cytokines such as IL-6, TNF α, interferon γ, IL-1 (28). An “autoimmune hypothesis” for short stature in CD has also been proposed (29), but evidences have been found only in few patients for whom high titers of anti-pituitary antibodies (APA) have been associated to low levels of IGF-1 (28). Hence APA titers might help identifying CD subjects with suspected GH deficiency (GHD).

The early introduction of GFD leads to a rapid growth catch-up, particularly in the first 6 months, with weight catch-up being much faster than height. Catch-up growth is a remarkable phenomenon characterized by an increase in height up to four times the average rate for the corresponding chronological age. Target height is usually reached within 3 years after diagnosis. However, sometimes CD patients do not reach their target height, possibly because a rapid catch-up growth can associate with accelerated bone maturation (27, 30, 31). When no catch-up growth can be observed despite a strict GFD, an endocrinological evaluation is mandatory to exclude GHD, condition that has been observed in ~0.23% of CD patients (32). Other comorbidities such as inflammatory bowel diseases (IBD), food aversion, Turner syndrome, have been reported in children with persistent short stature despite strict adherence to the GFD and should be ruled out in those cases. An early diagnosis and proper dietary regimen continuation minimize the risk of a compromised final height. Accordingly, Comba et al. (33) observed that patients who received the diagnosis of CD after the age of 6 had a significantly lower z-score for BMI, height and weight, as compared to children diagnosed at a younger age, indicating that when CD diagnosis is posed after puberty, the chances for growth catch-up are lower.

### Delayed Puberty

In the pediatric untreated CD population delayed puberty is a common finding, due to hypogonadism in girls and to androgen resistance in boys (34, 35). The prevalence of delayed puberty in CD is about 11–20% (36). The pathogenesis is unclear, however a combination of nutritional deficiencies and autoimmune antibodies against hormones, their receptors and/or endocrine organs, seem to play a role (34, 35, 37). The prognosis is favorable with puberty development occurring within 6–8 months from the introduction of GFD. If the delay in puberty persists, the patient should be referred to the endocrinologist for further evaluation.

### Anemia

Anemia is the most common extra-intestinal manifestation in the CD adult population, but roughly present in 15% of the CD pediatric population (10, 11, 19, 38), probably because in adults the diagnosis is delayed. The anemia is correlated with the severity of CD (39). In a recent meta-analysis, Mahadev et al. (40) observed that iron deficiency anemia is frequent in CD irrespectively of patient demographics (age and gender). Iron absorption occurs mainly in the duodenal mucosa, hence the small intestinal damage typical of active CD may lead to its malabsorption. However, the observation that anemia could be found also in children with potential celiac disease (41), seems to suggest a multifactorial pathogenesis. Anemia is most frequently due to iron deficiency, nevertheless vitamin B12 and folate deficiencies may also be responsible. Up to 84% of CD children presenting with mild anemia that strictly follow the GFD and receive iron supplementation show a complete recovery of their iron storages by 12–24 months, when their hemoglobin levels are not very low (10, 39). Oral iron formulations are often preferred over IV formulations. Recently, new oral iron formulations, sucrosomial iron, has been proposed for patients who are intolerant to iron sulfate.

### Liver Abnormalities

Hypertransaminasemia has been reported as the most frequent hepatic manifestation in CD patients. Recent studies report its prevalence at about 9–14% (42). Most times the liver damage is not severe and reversible, but in rare cases it can lead to liver failure (43). The grade of hypertransaminasemia is correlated to the duodenal mucosal damage, malabsorption, and serum levels of anti-endomysial and anti-tissue transglutaminase2 (TG2) antibodies. It has been hypothesized that the altered gut permeability can determine an increased exposure to hepatotoxins in the portal circulation leading to inflammation and liver damage (19, 44, 45). Nevertheless, in line with the other extra-intestinal manifestations, also in this case autoimmune factors may play a role, as indicated by the presence of anti-TG2 antibodies deposits in the liver (46).

The response of hypertransaminasemia to a strict GFD is excellent, with a 75–95% rate of complete normalization of liver enzymes in 12–24 months (47). An early diagnosis of CD may prevent future hepatic problems and a strict compliance of GFD can make unnecessary a routine control of liver function (42). A link between thyroid and liver disease has been reported, also based on the observation that usually CD patients with elevated ALT had also a higher TSH. The liver, in fact, performs a central role in the transport, metabolism, and deiodination of thyroid hormones (42).

As far as hepatological conditions associated with CD, primary biliary cholangitis, primary sclerosing cholangitis and autoimmune hepatitis have been found overrepresented in CD patients, but in contrast to the so called “coeliac hepatitis” (elevation of liver enzymes) their course is not modified by GFD.

### Bone Disease

Bone manifestations in CD patients are mainly osteopenia, defined as low bone mineral density (BMD), and osteoporosis, defined as low bone density leading to brittleness of the bones. Approximately 75% of pediatric patients have osteopenia and 10–30% osteoporosis (48). The damage of the duodenal mucosa, where both vitamin D and almost 90% of the calcium are absorbed, leads to decreased blood levels of calcium and vitamin D and consequent increased secretion of parathyroid hormone. The hyperparathyroidism is common in celiac children (12–54%) and it determines an increased bone turnover (49). Moreover, the inflammatory milieu including IL-1, TNFα, and IL-6, together with the activation of the RANK-L/RANK/osteoprotegerin pathway stimulates the bone metabolism (50, 51). The trabecular bone is usually the most involved, since it is the most metabolically active. Evaluation of the BMD is important in short-statured CD children, also in relation with height, gender, and age in order not to misinterpret BMD values. However, there is no demonstration of increased risk of fractures during childhood and youth in CD patients.

It is possible to have osteopenia also in the very early phases of the disease. A lower BMD has been observed in screening-detected CD children compared to controls; in the former, lower levels of 1-25-OH-Vitamin D and raised levels of PTH have also been measured. These values returned to the normal range after GFD was started, and that occurred already in the first year of GFD, emphasizing the importance of an early diagnosis (52, 53). Osteopenia may be further worsened by inadequate calcium intake, hence the need to supplement the GFD with Calcium-fortified foods and vitamin D metabolites (54, 55).

### Joint and Musculoskeletal Disorders

The most common joint and musculoskeletal disorders in CD include myopathy, arthralgia and non-erosive arthritis that can be silent in the early stages of disease (56). The most common finding in CD pediatric population is a subclinical synovitis, while arthralgia becomes evident with age (>12 years). The incidence of these manifestations is ~5–10% and the most commonly involved joint is the knee, followed by the hip and the ankle (19). Ultrasonography is important for the diagnosis, particularly in those patients whose symptoms are mild. The pathogenesis of the rheumatological manifestations remains obscure (56) and similarly their response to the GFD. Iqbal and colleagues reported an improvement only in 30% of patients (57) upon gluten withdrawal, suggesting that GFD may lead to improvement of symptoms at least in a subset of patients.

It is important to remember that CD can be associated with autoimmune diseases involving joints and muscles such as Sjogren's syndrome, juvenile idiopathic arthritis, rheumatoid arthritis and systemic lupus erythematosus (LES). A two-fold risk of LES has been recently reported in the pediatric celiac population (58).

### Neurological Manifestations

**Figure 1:**
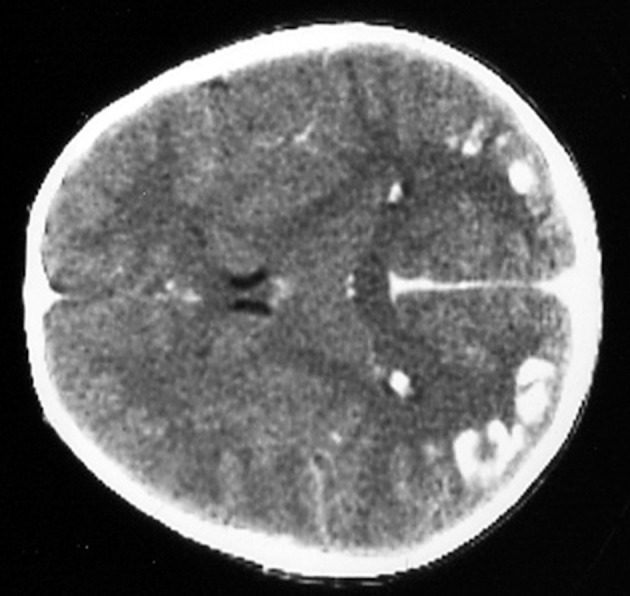
Bilateral occipital calcifications in celiac disease.

Several neurological manifestations are significantly associated with CD in the pediatric population. The most common being headache, that is present in up to one-fifth of the cases. Rarer conditions in the pediatric population are ataxia and neuropathy, ranging from 0.1 to 7.4%. A prevalence of 0.7–2%, not significantly different from the general population, has been described in some studies (59, 60), however other authors reported a 1.4 fold increase of epilepsy in CD children (61, 62). Thus, the link between epilepsy and CD remains still uncertain. The most common seizures patterns are the complex partial, followed by tonic-clonic seizures. A particular type of epilepsy characterized by the presence of occipital calcifications has been specifically reported in association to CD (63, 64)—Figure 1.

Cerebellar ataxia is more common in adults, with a median age of onset around 20 years. Chronic, symmetric distal neuropathy is the most common form of neuropathy described in CD patients, but its precise prevalence is unclear. A possible pathogenetic mechanism behind the neuropathy involves the anti-ganglioside antibodies, but nutritional deficiencies may also have a role. Particularly, some neurological manifestations are correlated with the deficit of vitamins such as E, B12 and D, or micronutrients like magnesium. Nevertheless, these manifestations may be present even in children with no enteropathy, excluding a role for malabsorption and suggesting that other mechanism might be responsible, for example a cross reaction between anti-gliadin antibodies and synapsin has been postulated. The pathogenesis of gluten ataxia can be related to the presence of anti-TG6 antibodies that might be directed against the cerebellar cells. However, their pathogenic role remains unclear since these autoantibodies can also be present in celiac children who are not affected by neurological disorders. The GFD leads to the complete recovery of headache in 76% of celiac children (65, 66) and it can be also responsible of the low prevalence of gluten ataxia and distal neuropathy when started early.

### Psychiatric Disorders

An association between CD and psychiatric disorders, including attention deficit and hyperactive disorder (ADHD), autism spectrum disorders (ASD), mood disorders, anxiety, eating disorders and depression, has been reported (67). In a large cohort of CD children, an increased risk of psychiatric disorders development (1.4-fold increase) has been observed (68). There is evidence supporting an association of CD with depression and, although to a less extent, with eating disorders (67). For panic disorder, autism and ADHD there are few reports indicating an association but further studies are necessary. Finally, the association between CD and schizophrenia or other anxiety disorders is still debated. As far as pathogenic mechanisms involved, a direct effect of CD, perhaps based on inflammation and immunological dysregulation has been proposed (69), but another possible concomitant cause could be the psychosocial discomfort associated with a chronic condition for CD children.

### Enamel Defects

The exact prevalence of enamel defects in CD is not known. However, in a recent meta-analysis it was observed that CD patients had significantly higher prevalence of enamel defects compared to controls (70). Some reports indicate that it involves up to 40–50% of CD patients at diagnosis (71), but in more recent reports the percentage results to be below 15% (72) probably because clinical presentations have become less severe over time. The enamel defects are characterized by pitting and sometimes by complete loss of enamel; they include discoloration and structural changes. Aine described enamel defects as detectable in all quadrants of the dentition, involving deciduous teeth (incisors and molars are the more frequently involved teeth) and most importantly symmetrical, the latter feature being more specific for CD (73). He proposed a grading, detailed in Table 1. Usually defects in dental enamel occur when CD affects children during dental development (before 7 years of age). When the defect affects permanent teeth, there is no improvement upon GFD.

**Table 1:** Classification of systemic and chronologic enamel defects [modified from Aine (74)].

**Grade 0**	**No defect**
Grade 1	Enamel discoloration with yellow, cream or brown opacities and loss of normal enamel glaze.
Grade 2	Structural defect with some horizontal grooves. Change of color can be find.
Grade 3	Important structural defects with deep horizontal grooves. Discoloration may be present.
Grade 4	Destruction of tooth shape and structure. The material of enamel is fragile.

**Figure 2:**
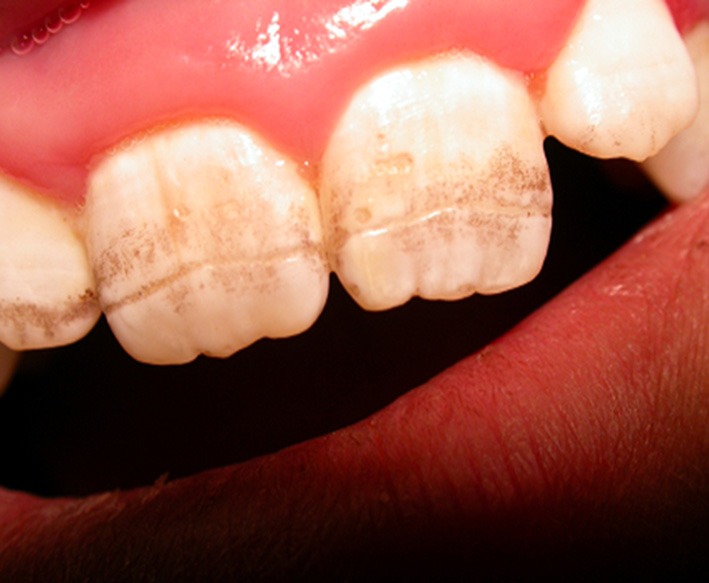
Model of enamel defects in celiac pediatric patient; these lesions have a symmetrical distribution.

The defect of amelogenesis, malnutrition, in particular hypocalcemia, and immunological disturbances have been proposed as causes of enamel defects in CD. However, some reports deny a correlation between the degree of enamel defects and the severity of small bowel mucosal damage (75). Patients with HLA-DR3 genotype have been reported to have a higher risk of enamel lesions, pointing to a genetic contribution (76) (Figure 2).

### Aphthous Stomatitis

Other oral disorders have been related to CD including delayed teeth eruption, lichen planus, cheilosis, atrophic glossitis, glossodinia. Aphthous stomatitis is an inflammatory ulcerative condition characterized by multiple recurrent small, round or ovoid ulcers with circumscribed margins appearing in the oral cavity. It usually manifests in the non-keratinized oral mucosa and can cause considerable pain. Up to 46% of CD patients have been reported to be affected by aphthous stomatitis (77). The mechanisms underlying this manifestation remain still obscure. It is unclear if there is any relation with malabsorption. Disturbances of the oral ecosystem (saliva, leukocytes, microbioma) have been hypothesized. Usually patients remit completely on GFD (10).

### Alopecia

Alopecia has been observed in ~1% of recently diagnosed CD children. Depending on the extension of the lesion it is possible to distinguish alopecia areata, totalis, and universalis. Autoimmune mechanisms are thought to be involved in its pathogenesis, nevertheless GFD can lead to the total regrowth of hair by 12–24 months in half of the cases (78).

### Dermatitis Herpetiformis

Dermatitis herpetiformis (DH) is considered an extraintestinal manifestation of CD. In children it is relatively rare (in Finland only 4% of all DH cases are children) (79), but in some series from other countries it seems to be more frequent in the pediatric age (80). It is particularly interesting to note that, in contrast to CD, the annual incidence rate is decreasing. It has been hypothesized that subclinical CD may predispose to DH, but what renders some individuals more prone to the development of skin lesions is unknown. The disease starts in the gut and evolves with the deposition in the papillary dermis of immune complexes of TG3 and high avidity anti-TG3 IgA antibodies (79).

From a clinical standpoint it presents with itchy papules and small blisters, often crusted because of the intense itch and consequent scratching, located on the extensor surfaces of elbows, knees and on the buttocks. Upper back, abdomen, scalp, and face may also be affected. Gastrointestinal symptoms in patients with DH are rare, but up to 72% of patients have a silent enteropathy (81). The diagnosis is made on a biopsy of unaffected skin showing by direct immunofluorescence pathognomonic granular IgA deposits at the dermo-epidermal junction. The rash recedes upon a strict GFD, with almost 100% resolution rate in children. Some patients may need additional medical therapy with dapsone, but with time the lesions are well-controlled by a GFD alone. In children the long-term prognosis on exclusion diet is excellent.

## Mechanisms underlying extra-intestinal manifestations

The pathogenesis of extra-intestinal manifestations in many respects is still unclear. There are two main mechanisms probably involved, one related to the mucosal damage and the consequent malabsorption, the second sustained by the autoimmune response. In line with the first, some extra-intestinal manifestations are clearly correlated with the severity of intestinal damage. Indeed, patients with extra-intestinal manifestations at diagnosis have a more severe grade of intestinal mucosal atrophy as compared to patients presenting only with gastrointestinal symptoms (11). Anemia is associated with malabsorption of iron, vitamin B12, and folate (82). Stunted growth is likely caused by nutrients malabsorption (25, 83). Finally, osteopenia may be due to malabsorption of calcium and vitamin D, leading to secondary hyperparathyroidism and subsequently a high bone turnover (84).

Extra-intestinal manifestations may also be the consequence of autoimmune phenomena, although in many cases this hypothesis needs to be supported by more evidences. Tissue-transglutaminase 2 (TG2) is the main, but not the only autoantigen involved in CD. Whether anti-TG2 autoantibodies play a role in CD pathogenesis has not been definitely proven. They bind to several epitopes including the enzymatic core and can then interfere with bioactivity of TG2 (85). The presence of IgA deposits co-localizing with TG2 in liver, lymphnodes, muscle, thyroid, bone and brain (86) indicate that the autoantibodies, probably originated in the gut, can access to TG2 throughout the body and cause pathogenic effects. Consistently with this hypothesis, data in mice showed that the injection of anti-TG2 antibodies in the lateral ventricle of the brain caused deficits in motor coordination (87). Other members of transglutaminase family possibly involved in the pathogenesis of extra-intestinal manifestations in CD are TG6 and TG3. The latter is mainly expressed in the cornified layer of epidermis and for this reason also defined epidermal TG. Antibodies anti-TG3 have been found in almost 95% of DH patients representing a useful diagnostic marker for DH in both pediatric and adult patients (88). They are present also in areas located far away from the skin lesions, suggesting that other factors, besides the mere presence of the antibodies, are necessary to provoke the lesions. The TG6 is mainly expressed in neurons, playing an important role in neurogenesis (89). An association between neurological manifestation in CD and the presence of anti-TG6 has been suggested. In fact, anti-TG6 is elevated in the serum of patients with gluten neuropathy (90). Furthermore, TG6 is the target autoantigen in gluten ataxia (91). However, both specificity of these autoantibodies and gluten-dependence of their production have not been definitely proven (92).

As said, tissue transglutaminase is not the only autoantigen in CD. Antibodies to gangliosides have been reported in immune mediated peripheral neuropathies (93, 94); in particular, they have been described in CD patients in conjunction with neurological symptoms (95), their titers responding to the exclusion of gluten from the diet (96). Neutralizing autoantibodies against osteoprotegerin have also been detected in CD patients (97), but their role in development of osteoporosis is still uncertain (98). Finally, autoantibodies to cardiolipin, enolase alfa, ATP synthase beta chain, and also IgA to collagen type I, III, V, VI have been reported in CD (99), but at present there is no association/correlation between these and extra-intestinal manifestations of CD.

We reported in Table 2 the possible pathogenetic mechanisms for each EIM in CD.

**Table 2:** Possible pathogenetic mechanisms for each extra-intestinal manifestations in celiac disease.

**Manifestation**	**Probable cause(s)**
**CUTANEOUS**	
Edema	Hypoproteinemia
Dermatitis herpetiformis	Epidermal (type 3) tTG autoimmunity
**ENDOCRINOLOGIC**	
Amenorrhea, delayed puberty	Malnutrition, hypothalamic-pituitary dysfunction, immune dysfunction
Secondary hyperparathyroidism	Calcium and/or vitamin D malabsorption with hypocalcemia
**HEMATOLOGIC**	
Anemia	Iron, folate, vitamin B12, or pyridoxine deficiency
Hemorrhage	Vitamin K deficiency
**HEPATIC**	
Elevated liver biochemical test levels	Celiac hepatitis
Autoimmune hepatitis	Autoimmunity
**MUSCULAR**	
Atrophy	Malnutrition due to malabsorption
Tetany	Calcium, vitamin D, and/or magnesium malabsorption
Weakness	Generalized muscle atrophy, hypokalemia
**NEUROLOGIC**	
Peripheral neuropathy	Deficiencies of vitamin B12 and thiamine; immune-based neurologic dysfunction
Ataxia	Cerebellar and posterior column damage
Demyelinating central nervous system lesions	Immune-based neurologic dysfunction
Seizures	Unknown
**SKELETAL**	
Osteopenia, osteomalacia, and osteoporosis	Malabsorption of calcium and vitamin D, secondary hyperparathyroidism, chronic inflammation
Pathologic fractures	Osteopenia and osteoporosis
**ORAL DISEASES**	
Enamel hypoplasia	Vitamin D, calcium malabsorption
Aphthous stomatitis	Unknown

## Treatment

Lifelong gluten-free diet (GFD) is the unique, effective therapy for CD (100). None of the pharmacological alternatives nowadays available or under investigation seems to be capable to replace the GFD (101).

In children GFD can lead to a complete recovery and a faster remission of extra-intestinal manifestations as compared to adults (10). The prognosis in children is very good, if adequately treated with GFD. However, timing of diagnosis is crucial. It is important to start the GFD as soon as possible. That means that an early diagnosis is decisive for a good prognosis. That is particularly true for bone diseases or for short stature. Patients diagnosed early during childhood did not have an increased risk of later development of osteoporotic fractures (102) and showed a very satisfactory catch up growth with a good final height (22). Nevertheless, sometimes the diet alone is not sufficient. This is the case of severe anemia, when it is advised to complement the dietary regimen with iron supplementation, or of severe DH, when adding a medical therapy with dapsone could be necessary in some cases (103), or even of severe osteopenia, when supplement the diet with calcium and vitamin D is important (54, 55). Problems arise when the GFD is not properly followed. This occurs mostly in adolescents (104), the pediatric population affected also by the highest prevalence of extra-intestinal manifestations and complications. When extra-intestinal manifestations show no improvement despite the GFD, patients' compliance to the dietary regimen should be questioned. In recent years a non-invasive, specific and novel approach to assess compliance with GFD has been reported, based on the detection of gluten immunogenic peptides (GIP) in the stools or in the urine by ELISA (105).

In case of no clinical improvement despite a strict GFD, additional diagnosis or pathogenic mechanism should be investigated. For instance, CD children presenting with short stature, but failing to show a satisfactory catch-up growth despite a verified compliance to the GFD, should be investigated for other co-existing conditions (e.g., growth hormone deficiency) (32) and consult the endocrinologist.

## Conclusions

With extra-intestinal manifestations dominating the clinical presentation of over half of the patients, also in children CD may be considered a systemic disease. These proteiform clinical features may complicate the diagnosis. With still too many cases being undetected, education becomes very important. Given the availability of very efficient non-invasive diagnostic tools, such as measurement of serum anti-TG2 antibodies, it is necessary to increase the awareness among general pediatricians, but also other specialists (hematologists, neurologists, rheumatologists, endocrinologists). A more careful case finding strategy, together with a more liberal use of CD-specific serological tests, will improve the detection rate. Furthermore, an early diagnosis is particularly important for a faster remission of symptoms, better prognosis as well as to prevent long-term complications, especially those that are no more correctable after a certain age (e.g., osteopenia, short stature). Finally, extra-intestinal manifestations may help understanding the pathogenic mechanisms of CD, in particular the role played by autoimmunity in the different clinical presentations.

## Author Contributions

SN: first draft of the manuscript, manuscript revision, final approval of the version to be published, and agreement to be accountable for all aspects of the work. RT: corresponding author, primary responsibility for communication with the journal during the manuscript submission, drafting the work, manuscript revision, final approval of the version to be published, agreement to be accountable for all aspects of the work. RA and VD: critical revision of the article, final approval of the version to be published, agreement to be accountable for all aspects of the work.

### Conflict of Interest Statement

The authors declare that the research was conducted in the absence of any commercial or financial relationships that could be construed as a potential conflict of interest.
